# Dynamic and Basal Phosphorylation Landscapes of Abscisic Acid Signaling Revealed by Phosphoproteome Analysis in Arabidopsis

**DOI:** 10.3390/ijms27083532

**Published:** 2026-04-15

**Authors:** Hinano Takase, Mizuki Saigusa, Kota Yamashita, Taishi Umezawa

**Affiliations:** 1 Graduate School of Bio-Applications and Systems Engineering, Tokyo University of Agriculture and Technology, Tokyo 184-8588, Japan; s255008q@st.go.tuat.ac.jp (H.T.); msaigusa@gmail.com (M.S.); ya.kouta.h071106@gmail.com (K.Y.); 2 Graduate School of Advanced Interdisciplinary Science, Tokyo University of Agriculture and Technology, Tokyo 184-8588, Japan; 3 Faculty of Agriculture, Tokyo University of Agriculture and Technology, Tokyo 183-8538, Japan

**Keywords:** phosphoproteome, ABA, *Arabidopsis thaliana*, SnRK2, PP2C

## Abstract

Abscisic acid (ABA) is a major phytohormone regulating plant growth and stress responses. Subclass III SnRK2 kinases and clade A type 2C protein phosphatases (PP2Cs) are core components of ABA signaling. Despite advances from phosphoproteomics, major gaps remain, particularly in mapping PP2C dephosphorylation targets and SnRK2-dependent phosphorylation dynamics under non-stress conditions. Here, we performed large-scale LC–MS/MS phosphoproteomic analyses using the subclass III SnRK2 triple mutant *srk2dei* and the constitutively active PP2C mutant *abi1–1C*, with and without ABA treatment in *Arabidopsis thaliana*. We identified 2757 and 2886 differentially regulated phosphopeptides in *srk2dei* and *abi1–1C*, respectively. Beyond known ABA signaling components, these datasets revealed numerous previously uncharacterized candidate proteins involved in metabolism, membrane transport, transcription, and cytoskeletal regulation. Integrative analysis uncovered a core set of candidate proteins oppositely regulated by SnRK2-mediated phosphorylation and ABI1-mediated dephosphorylation, defining a coordinated hierarchical network. These results indicate that the SnRK2–PP2C module functions not only in stress-induced ABA responses but also as a central regulator of phosphorylation homeostasis under basal conditions. This study provides a systematic framework for the global SnRK2–PP2C phosphorylation network and reframes ABA signaling as a dynamic homeostatic system.

## 1. Introduction

Abscisic acid (ABA) is a central phytohormone that regulates many aspects of the plant life cycle, including seed maturation, stomatal movement, and responses to both abiotic and biotic stresses [1,2,3]. A simple ABA signaling module consists of the PYR/PYL/RCAR family of ABA receptors, clade A type 2C protein phosphatases (PP2Cs), and SNF1-related protein kinase 2 (SnRK2) proteins [4]. In the absence of ABA, PP2Cs dephosphorylate and inhibit SnRK2 activity, thereby repressing ABA-dependent signaling pathways [5]. Environmental stresses or developmental cues lead to increased ABA accumulation in plants, and elevated ABA levels enable PYR/PYL/RCAR receptors to bind ABA and subsequently interact with and inhibit PP2Cs [6]. This inhibition releases SnRK2s from negative regulation, allowing their activation and the initiation of downstream ABA signaling [5,7].

In Arabidopsis, there are 10 SnRK2 genes, grouped into subclasses I, II, and III [8]. These kinases are activated by ABA and/or osmotic stress, so they function in both ABA-dependent and ABA-independent stress pathways [8,9]. Three subclass III members—SRK2D/SnRK2.2, SRK2E/OST1/SnRK2.6, and SRK2I/SnRK2.3—are strongly activated by ABA and osmotic stress and regulate many stress-related processes [5,10]. SRK2E mainly functions in guard cells to regulate stomatal opening and closing, whereas SRK2D and SRK2I contribute to ABA responses during seed germination, dormancy, and early seedling growth [11,12,13]. Several downstream targets of SnRK2s have been identified, including the AREB/ABF transcription factors, whose phosphorylation is essential for ABA-responsive gene expression [14,15]. SnRK2s also phosphorylate membrane proteins such as SLAC1, RbohF, and KAT1, modulating stomatal closure and other stress-related physiological responses [16,17,18,19]. Plants lacking all three genes (the *srk2dei* mutant) show an extremely weak response to ABA, indicating that these kinases function redundantly and form the core of ABA signaling [5,20].

Clade A PP2C includes 9 members in Arabidopsis, which can be further divided into two subfamilies based on sequence similarity: one comprising ABA insensitive 1 (ABI1), ABI2, hypersensitive to ABA 1 (HAB1), and HAB2, and the other comprising ABA hypersensitive germination 1 (AHG1), AHG3, highly ABA-induced PP2C gene 1 (HAI1), HAI2, and HAI3 [21,22]. At least six clade A PP2Cs, including ABI1, ABI2, HAB1, HAB2, AHG1, and AHG3, act as negative regulators of ABA-dependent SnRK2 activation [5,7,23]. ABI1 is one of the key PP2Cs in ABA signaling, and its gain-of-function mutant *abi1-1C* exhibits a strong ABA-insensitive phenotype [5]. Because ABI1 is thought to regulate additional targets beyond SnRK2s, the full extent of PP2C-mediated control remains unclear.

As described above, protein phosphorylation plays a central role in abscisic acid (ABA) signaling. Protein phosphorylation is a major post-translational modification that regulates diverse biological processes, including signal transduction, transcriptional control, and metabolic regulation. In plants, phosphorylation is particularly essential for coordinating responses to environmental stresses and hormonal cues. Recent advances in mass spectrometry have enabled comprehensive phosphoproteomic analyses, allowing large-scale identification and quantification of phosphorylation sites at the proteome level. This approach provides a powerful means to capture global signaling architectures that are difficult to resolve using conventional analyses focused on individual components. Moreover, the integration of time-resolved phosphoproteomics with analyses across distinct genetic backgrounds enables detailed dissection of the dynamics and hierarchical organization of phosphorylation regulation. Although phosphoproteomic analyses have been applied to plant hormone signaling, particularly ABA responses, and have revealed parts of the signaling network, the complete landscape and temporal control mechanisms of ABA-dependent phosphorylation remain incompletely understood.

To gain a comprehensive understanding of ABA-dependent phosphorylation networks, we performed comparative phosphoproteomic analyses using wild type (Col-0) and two types of ABA-insensitive mutants: *srk2dei* and *abi1–1C*. This approach enabled the identification of phosphorylation events that depend on SnRK2 activity, PP2C activity, or both, providing new insights into the molecular logic underlying ABA signaling.

## 2. Results and Discussion

### 2.1. Overview of Phosphoproteome Analysis

**Figure 1:**
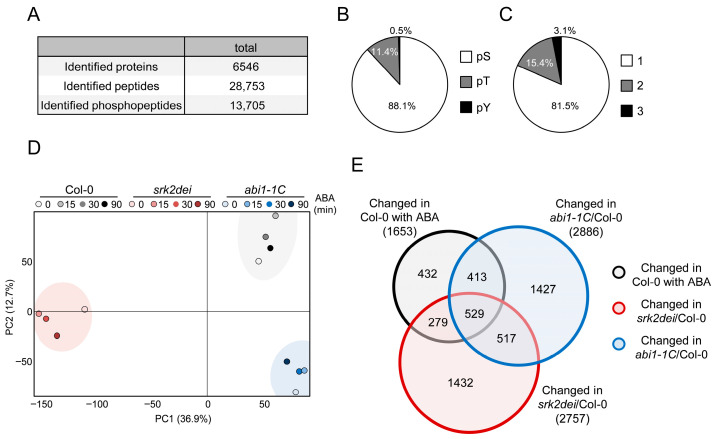
Overview of the phosphoproteomic data from Col-0, *srk2dei* and *abi1–1C*. (**A**) Numbers of identified proteins, peptides and phosphoproteins detected by LC-MS/MS. (**B**) Distribution of phosphorylated residues in each peptide. (**C**) Distribution of the number of phosphosites per peptide. (**D**) Principal component analysis (PCA) of the phosphoproteomic data. (**E**) Venn diagram showing the overlap between ABA-responsive phosphopeptides in Col-0 and phosphopeptides up- or down-regulated phosphopeptides in *srk2dei* or *abi1–1C*.

For phosphoproteome analysis, two-week-old *Arabidopsis thaliana* (L.) Heynh. Col-0, *srk2dei*, and *abi1–1C* seedlings were treated with 50 µM ABA, and samples were collected at 0, 15, 30, and 90 min after treatment. On average, approximately 22,000 phosphorylated peptides were identified per sample (Figure 1A, Supplementary Figure S1). Among the identified phosphorylation sites, 88.1% were localized to serine (Ser) residues, 11.4% to threonine (Thr) residues, and 0.5% to tyrosine (Tyr) residues, indicating that phosphorylation predominantly occurred on Ser and Thr residues (Figure 1B). In addition, the proportions of peptides containing one, two, or three phosphorylation sites were 81.5%, 15.4%, and 3.1%, respectively (Figure 1C). These distributions are broadly consistent with those reported in previous phosphoproteome studies in plants [24,25].

Principal component analysis (PCA) of Col-0 samples revealed clear separation between ABA-treated samples (15, 30, and 90 min) and untreated controls (Supplementary Figure S2), indicating that ABA treatment effectively induced substantial changes in phosphorylation profiles. In contrast, PCA of all samples showed that Col-0, *srk2dei*, and *abi1–1C* formed distinct clusters, suggesting that genetic background strongly influences global phosphorylation patterns (Figure 1D).

In Col-0, 1653 phosphopeptides exhibited significant changes in abundance following ABA treatment, of which 1221 showed significant differences in either *srk2dei* or *abi1–1C* (Figure 1E). A guard cell-specific phosphoproteome study reported in 2025 identified 1894 ABA-responsive phosphopeptides in Col-0, of which 1202 showed significant changes in *srk2de* or *abi1–1C* [26]. The results obtained in the present study show strong quantitative agreement with this previous work. Furthermore, comparison of ABA-responsive phosphoproteins identified in wild-type plants in this study (1136 genes) with those reported in the guard cell protoplasts (GCPs) analysis (1352 genes) revealed 260 genes in common (Supplementary Figure S3A). Similar comparisons for phosphorylated proteins altered in *srk2dei* and *abi1–1C* identified 934 and 838 overlapping genes, respectively (Supplementary Figure S3B,C). In addition, analysis of ABA-responsive phosphopeptides in Col-0 that were altered in both t *srk2dei* and *abi1–1C*—representing factors likely subject to SnRK2-dependent phosphorylation—identified 121 common genes (Supplementary Figure S3D).

Network analysis of ABA-responsive phosphoproteins revealed distinct temporal patterns. During the 15–30 min window following ABA treatment, a network centered on SnRK2 was observed, which included phosphorylation changes in PHOT and AHA (Supplementary Figure S4). In contrast, during the 30–90 min time window, a network centered on MAPK became prominent. These results suggest that, under the experimental conditions used in this study, SnRK2 activity is dominant during the early phase of ABA treatment and diminishes at later stages.

### 2.2. Screening for SnRK2-Regulated Proteins

**Figure 2:**
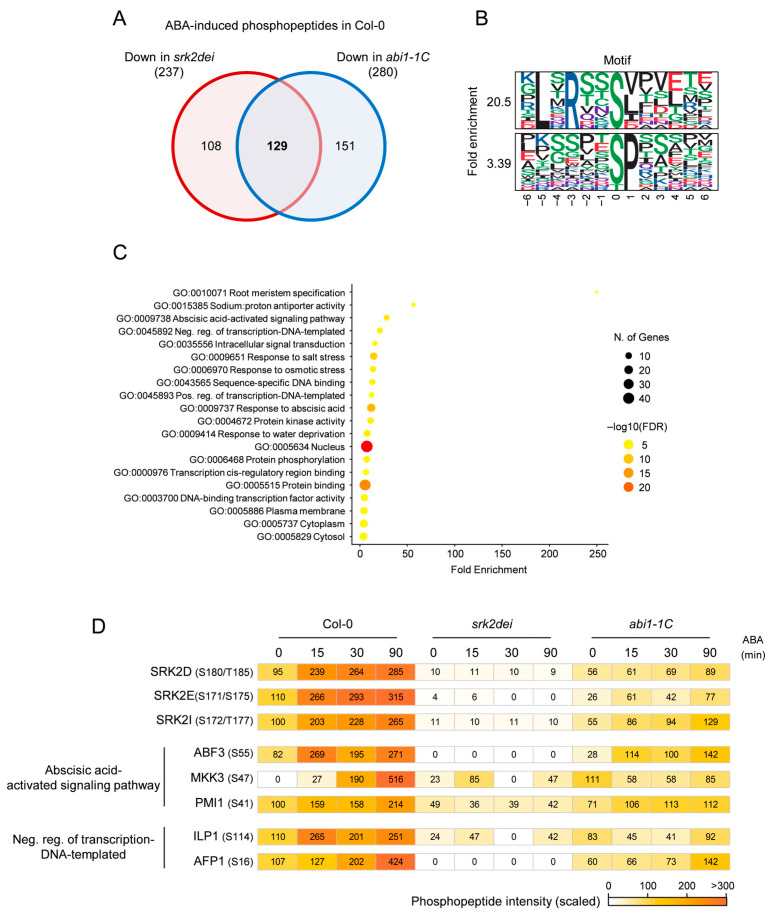
Phosphoproteome-based profiling of SnRK2-regulated proteins. (**A**) Venn diagram showing the overlap between phosphopeptides downregulated in *srk2dei* or *abi1–1C* in ABA-responsive phosphopeptides in Col-0. (**B**) Motif analysis of downregulated phosphopeptides in both *srk2dei* and *abi1–1C* (fold change < 0.5, *p* < 0.05). Two major motif groups were identified: Group 1, [-L-x-R-x-x-pS/pT-]; Group 2, [-pS/pT-P-]. (**C**) GO analysis of downregulated phosphopeptides in both *srk2dei* and *abi1–1C* by shinyGO program (fold change < 0.5, *p* < 0.05). (**D**) Heat map showing the intensities of phosphopeptides from known SnRK2-regulated proteins. To compare peptide abundance across conditions, mean intensities were scaled to the same range for each phosphopeptide.

To identify SnRK2-regulated proteins, we extracted phosphopeptides whose abundance was significantly increased by ABA treatment in Col-0 but reduced in *srk2dei* or *abi1–1C*, and compared these datasets using a Venn diagram (Figure 2A). As a result, 129 phosphopeptides were found to be decreased in both *srk2dei* and *abi1–1C*. After excluding three phosphopeptides derived from SnRK2 itself, the remaining 126 phosphopeptides were selected as candidate downstream of SnRK2.

These candidate proteins included bZIP-type transcription factors, MAP kinases, VCS, SNS1, and MBD10, all of which have previously been reported to be directly or indirectly regulated by SnRK2-dependent phosphorylation, thereby supporting the reliability of this analysis (Supplementary Figure S5) [10]. Motif analysis of the 129 selected phosphopeptides revealed significant enrichment of both the [-R-x-x-pS/pT-] motif, which is characteristic of SnRK2 substrates, and the [-pS/pT-x-] motif, known as a target motif of MAPKs (Figure 2B). Notably, similar ABA-induced enrichment of the SP and RxxS motifs has also been reported in rice and soybean phosphoproteomes [27], suggesting that these phosphorylation signatures may be conserved across diverse plant species.

Gene Ontology (GO) analysis of the 111 genes encoding these phosphopeptides showed significant enrichment of the biological processes “abscisic acid-activated signaling pathway” and “Neg. reg. of transcription-DNA-templated” (Figure 2C). The relative phosphorylation levels of proteins belonging to these GO categories are presented as a heatmap (Figure 2D). The “abscisic acid-activated signaling pathway” category included genes such as ABF3 and MKK3. bZIP-type transcription factors of the AREB/ABF family are well established as direct substrates of SnRK2 and function as central regulators of ABA-responsive gene expression [28]. Phosphorylation of ABF3 at S55, identified in this study, has been reported to modulate its transcriptional activity, and this site is highly conserved from algae to higher plants [10,27].

In contrast, MKK3 has been reported to negatively regulate salt stress responses through activation of the transcription factor MYC2 via MPK6 [29,30,31]. MYC2 is a master transcription factor of jasmonate signaling and plays a key role in coordinating plant growth, defense, and metabolism [32]. In addition, MYC2 serves as a key integrator of multiple hormone signaling pathways, including ABA. For example, MYC2 enhances ABA2 expression, and loss of MYC2 disrupts ABA-responsive transcriptional patterns, supporting its role in linking JA and ABA signaling [33]. The phosphorylation dynamics observed in this study are consistent with these earlier findings.

The “Neg. reg. of transcription-DNA-templated” category included factors such as AFP. The AFP family functions as negative regulators of ABA signaling by interacting with ABI5 and suppressing its activity and stability [33]. In this study, AFP1 was found to be phosphorylated at three sites (S16, S75, and S115) in an ABA-responsive and SnRK2/ABI1-dependent manner. AFP proteins have also been reported to interact with SnRK1α, suggesting that they may act as integrators of multiple phosphorylation-dependent signaling pathways [34].

### 2.3. Screening for ABI1-Mediated Dephosphorylation

**Figure 3:**
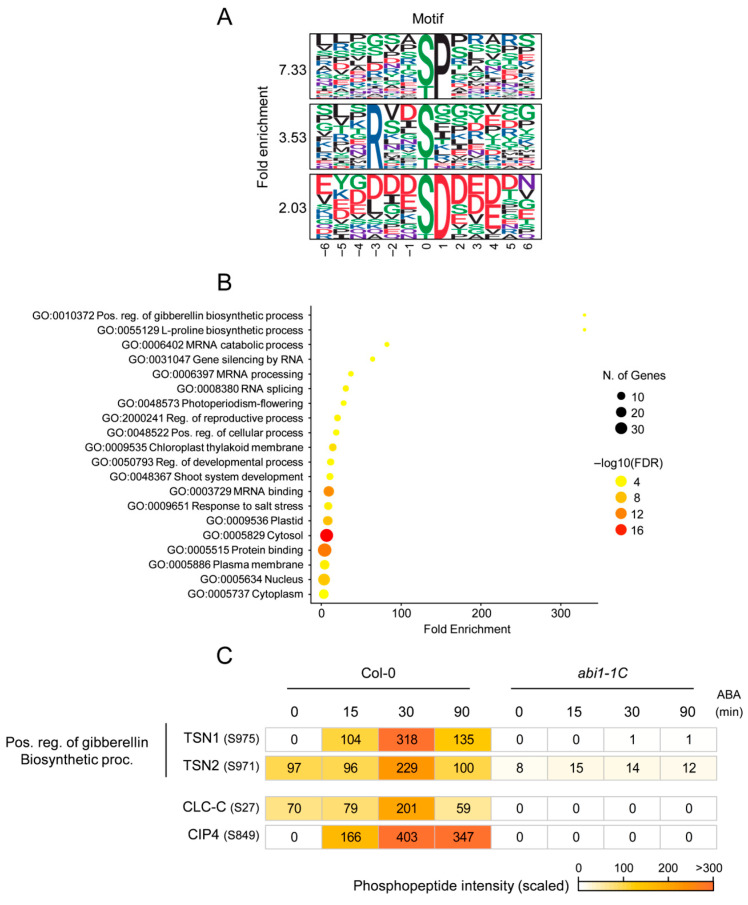
Phosphoproteome-based profiling of ABI1-regulated proteins. (**A**) Motif analysis of phosphopeptides downregulated in *abi1–1C* but not in *srk2dei* (fold change < 0.5, *p* < 0.05). Three major motif groups were identified: Group 1, [-pS/pT-P-]; Group 2, [-R-x-x-pS/pT-]; Group 3, [-pS/pT-D-]. (**B**) Gene Ontology (GO) analysis of phosphopeptides downregulated in *abi1–1C* but not in *srk2dei* using shinyGO program (fold change < 0.5, *p* < 0.05). (**C**) Heat map showing the intensity of phosphopeptides from ABI1-regulated proteins. To compare the peptide abundance between samples under different conditions, the mean intensities were scaled to the same range for each phosphopeptide.

To identify SnRK2-independent pathway regulated by ABI1-mediated dephosphorylation, we extracted phosphopeptides that were significantly increased by ABA treatment in Col-0 but specifically decreased in *abi1–1C* (Figure 2A). This analysis identified 151 candidate phosphopeptides. Motif enrichment analysis revealed significant overrepresentation of the [-pS/pT-P-], [-R-x-x-pS/pT-], and [-pS/pT-D-] motifs (Figure 3A).

GO enrichment analysis indicated significant enrichment of “positive regulation of gibberellin biosynthetic process,” and “L-proline biosynthetic process” (Figure 3B). The gibberellin-related category included TSN1 and TSN2, in which phosphorylation at S975 and S971, respectively, increased upon ABA treatment and decreased in *abi1–1C* (Figure 3C). Given the proposed role of TSN proteins in stress granule formation, these phosphorylation events may contribute to stress granule dynamics [35,36].

We also compared the 140 candidate ABI1-regulated proteins identified in this study with 1542 ABI1-interacting proteins reported in a previous TurboID-based study (Supplementary Figure S6) [37]. This comparison revealed 42 overlapping proteins. Among these were TSN2 and three aluminum-induced proteins containing YGL and LRDR motifs, including MQD19.19.

### 2.4. Screening for SnRK2-Regulated Proteins Under Basal ABA Conditions

Even under non-stress conditions, basal ABA levels are essential for maintaining plant growth and water homeostasis [38]. The drought-sensitive and growth-defective phenotype of the *srk2dei* mutant under normal conditions suggests that SnRK2 activity supported by basal ABA is required for normal development [9,39]. Furthermore, recent biosensor-based assays have demonstrated that SnRK2 possesses stress-independent kinase activity [40]. This notion is supported by the clear separation of Col-0 and *srk2dei* in PCA under ABA-free conditions (Figure 1C). However, few studies have focused on and investigated SnRK2-mediated phosphorylation events under normal conditions.

To screen for SnRK2-mediated phosphorylation under basal ABA conditions, we compared phosphoproteomic profiles of Col-0 and *srk2dei* in the absence of ABA. This analysis identified 574 phosphopeptides with increased abundance and 556 with decreased abundance in *srk2dei*.

**Figure 4:**
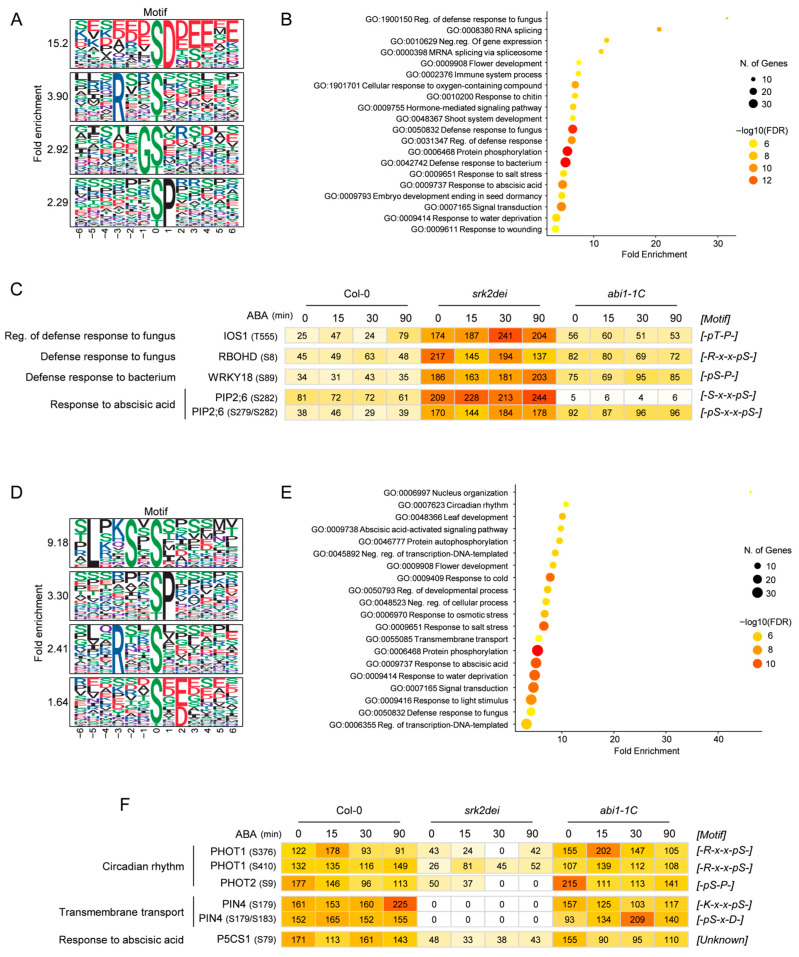
Basal ABA-dependent profiling of SnRK2-regulated proteins. (**A**) Motif analysis of phosphopeptides upregulated in *srk2dei* under control conditions (fold change > 2, *p* < 0.05). Four major motif groups were identified: Group 1, [-pS/pT-D-]; Group 2, [-R-x-x-pS/pT-]; Group 3, [-G-pS/pT-]; Group 4, [-pS/pT-P-]. (**B**) Gene Ontology (GO) analysis of phosphopeptides upregulated in *srk2dei* under control conditions using shinyGO program (fold change > 2, *p* < 0.05). (**C**) Heat map showing the intensities of phosphopeptides upregulated in *srk2dei* under control conditions. Mean intensities were scaled to the same range for each phosphopeptide to allow comparison across conditions. (**D**) Motif analysis of phosphopeptides downregulated in *srk2dei* under control conditions (fold change < 0.5, *p* < 0.05). Four major motif groups were identified: Group 1, [-L-x-x-S-x-pS/pT-]; Group 2, [-pS/pT-P-]; Group 3, [-R-x-x-pS/pT-]; Group 4, [-pS/pT-x-E/D-]. (**E**) Gene Ontology (GO) analysis of phosphopeptides down-regulated in *srk2dei* under control conditions using shinyGO program (fold change < 0.5, *p* < 0.05). (**F**) Heat map showing the intensities of phosphopeptides downregulated in *srk2dei* under control conditions. Mean intensities were scaled to the same range for each phosphopeptide, as in (**C**).

Up-regulated peptides in *srk2dei* were enriched for the [-pS/pT-D-], [-R-x-x-pS/pT-], [-G-pS/pT-], and [-pS/pT-P-] motifs (Figure 4A). GO analysis revealed that up-regulated phosphopeptides were enriched in terms such as “immune system process”, “defense response to fungus”, and “defense response to bacterium” indicating increased phosphorylation of genes involved in biotic stress responses (Figure 4B). Interestingly, genes enriched in the “Regulation of/Defense response to fungus” GO term included the receptor-like kinase IMPAIRED OOMYCETE SUSCEPTIBILITY 1 (IOS1) and the NADPH oxidase RBOHD, while “Defense response to bacterium” included various factors involved in immune responses, such as the transcription factor WRKY18 (Figure 4C). IOS1 is a negative regulator of ABA responses but acts as a positive regulator of pattern-triggered immunity (PTI) responses, contributing to the activation of MPK3/6 [41,42]. RBOHD is the primary source of reactive oxygen species (ROS) production during immune and ABA responses, and its activity is regulated by phosphorylation [43,44,45]. WRKY18 is a negative regulator of ABA responses and it is phosphorylated by MPK3/6 and positively regulates effector-triggered immunity (ETI) by transcribing the protein phosphatases AP2C1/PP2C5 [46,47]. Although the specific WRKY18 phosphorylation sites targeted by MPK3/6 remain unknown, S89 is a likely candidate given its SP motif. This correlates with previous research suggesting that ABA-mediated abiotic stress responses and biotic stress responses, such as disease resistance, often can act antagonistically [48].

Furthermore, PIP2;6 was enriched in the “Response to abscisic acid” GO term, with phosphorylation at S279 and S282 increased in srk2dei (Figure 4C). Our phosphoproteomic data further showed that phosphorylation at corresponding sites in PIP2;5, PIP2;7, and PIP2;8 was also elevated in *srk2dei* (Supplementary Table S1). In guard cells, SRK2E/OST1 is known to phosphorylate Ser-121 of PIP2;1, promoting water efflux from cells and ROS influx into cells [49,50]. Our results suggest the possibility that SnRK2 contributes to the phosphorylation of other PIPs, such as PIP2;6, even under normal conditions. Studies on PIP2;1 indicate that phosphorylation at positions corresponding to S279 and S282 in PIP2;6 is required for the plasma membrane localization of PIP2;1 and is critical for 14–3-3 protein binding and subsequent water transport activity [51,52]. Furthermore, it has been suggested that the phosphorylation sites of S280 (S273) and S283 (S276) in PIP2;1 (PIP2;7) are regulated by SnRK1-dependent phosphorylation under submergence [53].

Under normal conditions, Arabidopsis forms a PP2C-SnRK1/2 phosphatase-kinase complex that sequesters SnRK1 from the cytoplasm into the nucleus, thereby inhibiting its activity [54,55]. It is possible that PIP2;5–8, identified in this study, are phosphorylated by SnRK1 that has translocated to the cytoplasm after being released from the PP2C-SnRK1/2 complex due to the loss of SnRK2. Together, our phosphoproteomic observations suggest potential regulatory connections involving IOS1 and PIP2;6; however, biochemical validation will be required to determine whether these proteins are direct targets of SnRK2 or other kinases.

Down-regulated peptides in *srk2dei* were enriched for the [-L-x-x-S-x-pS/pT-], [-pS/pT-P-], [-R-x-x-pS/pT-], and [-pS/pT-x-E/D-] motifs (Figure 4D). GO analysis revealed that down-regulated phosphopeptides were enriched in terms such as “Leaf development” and “Circadian rhythm,” which is consistent with previous reports that *srk2dei* exhibits an increased number of leaves and earlier flowering compared to the wild type (Figure 4E) [39,56]. Phosphorylation of the blue light (BL)-receptor kinases PHOT1 and PHOT2, enriched in the “Circadian rhythm”, was reduced in *srk2dei* under normal conditions. The decreased p-sites of S376 and S410 in the hinge region of PHOT1 are phosphorylated in a BL-dependent manner and necessary for infighureteraction with 14–3-3 proteins [57,58]. While PHOT2 lacks this 14–3-3 binding site in its hinge region [59], phosphorylation at S9 at the N-terminus was decreased in *srk2dei*. Although the impact of S9 phosphorylation on PHOT2 function remains unclear, these results suggest that SnRK2 may be involved in regulating the phosphorylation of PHOT1/2 and their signaling components under normal conditions.

Additionally, the “Transmembrane transport” term included several PIN-FORMED (PIN) proteins. A recent report indicated that in Arabidopsis roots, PID and SRK2D/I (SnRK2.2/2.3) competitively phosphorylate adjacent sites S258 and S259 of PIN2, respectively, to regulate PIN2 polarity [60]. Conversely, S259 of PIN2, targeted by SRK2D/I, is conserved only in PIN1 [61]. Our phosphoproteomic analysis revealed that phosphorylation of the S179 and S183 residues of PIN4 was completely abolished in *srk2dei* (Figure 4F). Phosphorylation sites showing similar trends were also identified in PIN3 and PIN7 (Supplementary Table S1). The phosphorylation sites corresponding to PIN4 S179 and S183 are conserved across PINs except for PIN6 [61]; these results suggest that SnRK2 may regulate the phosphorylation of PIN proteins other than PIN2 under normal conditions.

Furthermore, the “Response to abscisic acid” term included P5CS1, a proline biosynthesis enzyme, and phosphorylation of P5CS1 S79 was decreased in *srk2dei* (Figure 4F). P5CS1 contains ABREs in its promoter region, and its transcription is upregulated in an ABA-responsive manner via ABFs [62,63]. Indeed, metabolomic analysis of srk2dei has shown that proline levels are reduced even under steady-state conditions [39]. Our findings suggest that SnRK2 may induce proline synthesis via phosphorylation of P5CS1 under normal conditions.

Interestingly, the phosphorylation motifs enriched under basal conditions closely resemble those observed during the dynamic ABA response (Figure 2B and Figure 4D), indicating that the fundamental substrate preferences of SnRK2 do not differ markedly between these states. This similarity suggests that basal SnRK2 activity may phosphorylate many of the same targets as stress-induced SnRK2 activation, potentially supporting growth and homeostasis under non-stress conditions.

## 3. Materials and Methods

### 3.1. Plant Materials and Growth Conditions

*Arabidopsis thaliana* (L.) Heynh. ecotype Columbia-0 (Col-0) seeds were obtained from our laboratory stock, originally sourced from Lehle Seeds Inc. (Round Rock, TX, USA). Sterilized seeds were sown on GM medium containing 1% sucrose and solidified with 0.8% agar. After 4 days of dark stratification at 4 °C, seedlings were grown for 2 weeks under long-day conditions (16 h light/8 h dark, 22 °C) with white LED illumination at 90 µmol m^−2^ s^−1^. ABA (50 µM) was applied through the roots, and tissues were collected at 0, 15, 30, and 90 min. Samples were immediately frozen and stored at −80 °C.

### 3.2. Sample Preparation for Phosphoproteomic Analysis

Protein extraction and digestion were performed essentially as described previously, with minor modifications [26,27]. Proteins were extracted from plant tissues using an extraction buffer containing 100 mM Tris-HCl (pH 9.0) and 6 M guanidine hydrochloride (Gdn-HCl). The extracts were heated at 95 °C for 5 min and subsequently subjected to methanol–chloroform precipitation. The resulting protein pellets were dissolved in a digestion buffer composed of 100 mM Tris-HCl (pH 9.0), 12 mM sodium lauroyl sarcosinate (SLS), and 12 mM sodium deoxycholate (SDC). A total of 400 µg of protein was then reduced and alkylated, followed by overnight digestion at 37 °C using 2 µg of trypsin (Promega, Madison, WI, USA). Phosphopeptides were enriched using hydroxy acid–modified metal oxide chromatography (HAMMOC) as described previously [64]. Digested peptides or enriched phosphopeptides were desalted using in-house StageTips packed with SDB Empore disks (CDS). After desalting, phosphopeptides were dried and stored at −80 °C until LC–MS/MS analysis.

### 3.3. LC-MS/MS Analysis and Raw Data Processing

Peptide samples were analyzed using an Easy-nLC 1200 system coupled to an Orbitrap Exploris 480 mass spectrometer equipped with a FAIMS Pro interface, essentially as described previously [26,27]. Dried peptide samples were resuspended in 2% acetonitrile containing 0.1% formic acid and injected onto a C18 nano-HPLC column. Peptide separation was performed with a 140-min nonlinear gradient at a flow rate of 300 nL/min, followed by ionization using nano-electrospray ionization (nano-ESI). Mass spectrometric data were collected in positive ion mode under data-dependent acquisition settings.

The raw MS data were analyzed using Proteome Discoverer version 2.5. Peptide identification was carried out with the SEQUEST HT search algorithm, and peptide–spectrum matches were filtered to achieve a false discovery rate (FDR) below 1% using Percolator. Protein inference and MS1-based label-free quantification were conducted following the workflow described previously [26,27].

### 3.4. Analysis of Proteomic Data

Unless otherwise noted, basic calculations related to proteomic data were performed in Excel program. GO enrichment analysis was performed as follows: the protein IDs were uploaded to ShinyGO 0.85.1 (https://bioinformatics.sdstate.edu/go/, accessed on 10 April 2026).

Motif analysis was performed as follows: The phosphorylation sites of phosphopeptides detected by Proteome Discoverer were centered, and the 6 amino acids upstream and downstream were extracted as foreground sequences. As background sequences, the 6 amino acids upstream and downstream of serine, threonine, and tyrosine residues in all registered genes of the corresponding plant species were extracted. Motif creation utilized rmotifex and ggseqlogo package from the motifR package (https://github.com/wangshisheng/motifeR (accessed on 10 January 2026)). The R script used is available at https://github.com/sou-06/Phospho_Logo_PD (accessed on 10 January 2026).

For network analysis, edges and confidence scores were generated based on protein–protein interaction information provided by PhosPhAt 4.0 (https://phosphat.uni-hohenheim.de/ (accessed on 10 January 2026)) and STRING (https://string-db.org/ (accessed on 10 January 2026)). We selected edges with confidence scores greater than 0.4. Directed edges were based on the interaction types “Phosphorylation” or “Activation” in PhosPhAt 4.0. Undirected edges were based on “interaction” in PhosPhAt 4.0 or interaction information from STRING. Cytoscape v3.10.4 (https://cytoscape.org/) was used for network visualization. To construct multi-layer networks, we used the TimeNexus app on Cytoscape with default settings, following the methods of a previous study [65]. Following that study, we extracted active subnetworks using PathLinker. For each time point, the value of the phosphorylation site exhibiting the maximum or minimum log2 FC was adopted as the representative value for the node. To extract subnetworks, we defined nodes satisfying |log2 FC| ≥ 1 and *p* < 0.01 as queries. We set the k-value to 250 and used the pairwise method for comparisons between layers in PathLinker.

## 4. Conclusions

This study substantially expands the ABA-responsive phosphoproteome in *A. thaliana*, providing higher-resolution insights into phosphorylation dynamics and revealing that distinct regulatory factors operate at different response stages of the ABA response. Phosphoproteomic analyses using SnRK2 and PP2C mutants demonstrated that diverse processes, including transcriptional regulation, stress granule formation, and metabolic control, are involved in ABA signaling. Notably, differences in phosphorylation states were observed even under control conditions, suggesting that basal ABA levels contribute to plant growth and cellular homeostasis. A limitation of this study is that the analyses were performed using whole seedlings, which does not allow the resolution of tissue-specific phosphorylation events. Future functional studies of the identified phosphoproteins will be required to elucidate the physiological and molecular roles of individual phosphorylation events, thereby advancing our understanding of the temporal and hierarchical mechanisms underlying ABA signaling.

## Data Availability

LC-MS/MS raw data of phosphoproteomic analysis have been deposited in Japan Proteome Standard Repository/Database (jPOSTP) [66]. Phosphoproteomics; link (https://repository.jpostdb.org/preview/5144600446989ce44c9f15 (accessed on 1 February 2026)), Access key (5215).

## Supplementary Materials

The following supporting information can be downloaded at: https://www.mdpi.com/article/10.3390/ijms27083532/s1.
