# Supplementary material for: Dynamic and Basal Phosphorylation Landscapes of Abscisic Acid Signaling Revealed by Phosphoproteome Analysis in Arabidopsis
Source: Int J Mol Sci. 2026 Apr 15;27(8):3532. doi: 10.3390/ijms27083532 (PMC13116211; doi:10.3390/ijms27083532)
Supplement: Supplementary file 1 [file ijms-27-03532-s001.zip › 260304_Sup_嶳壓_HT_TU.pptx]

## Slide 1
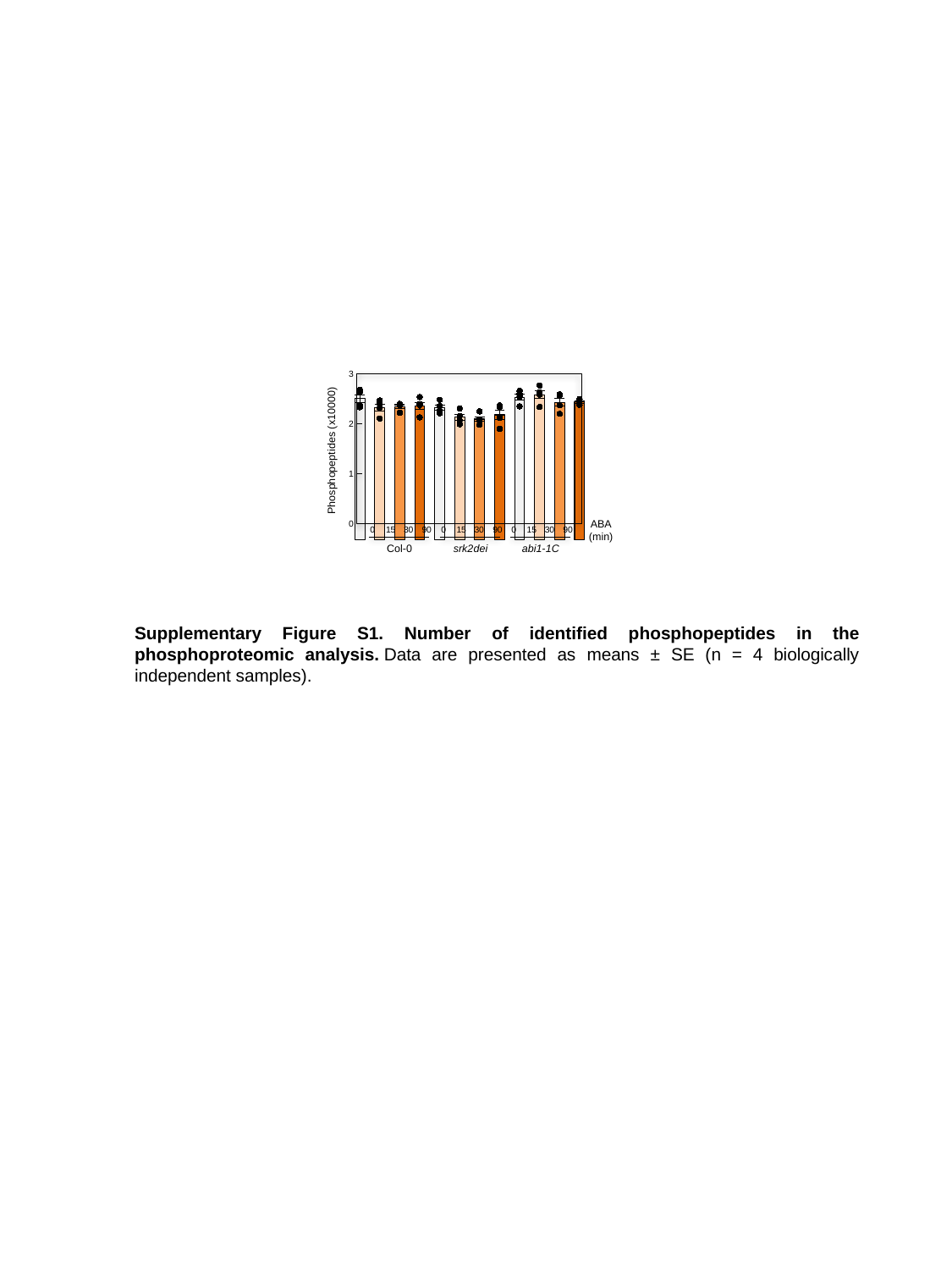

### Chart
| Category | | | | | |
|---|---|---|---|---|---|3
2
1
0
Phosphopeptides (x10000)
ABA
(min)
0
15
30
90
0
15
30
90
0
15
30
90
Col-0
srk2dei
abi1-1C
Supplementary Figure S1. Number of identified phosphopeptides in the phosphoproteomic analysis. Data are presented as means ± SE (n = 4 biologically independent samples).

## Slide 2
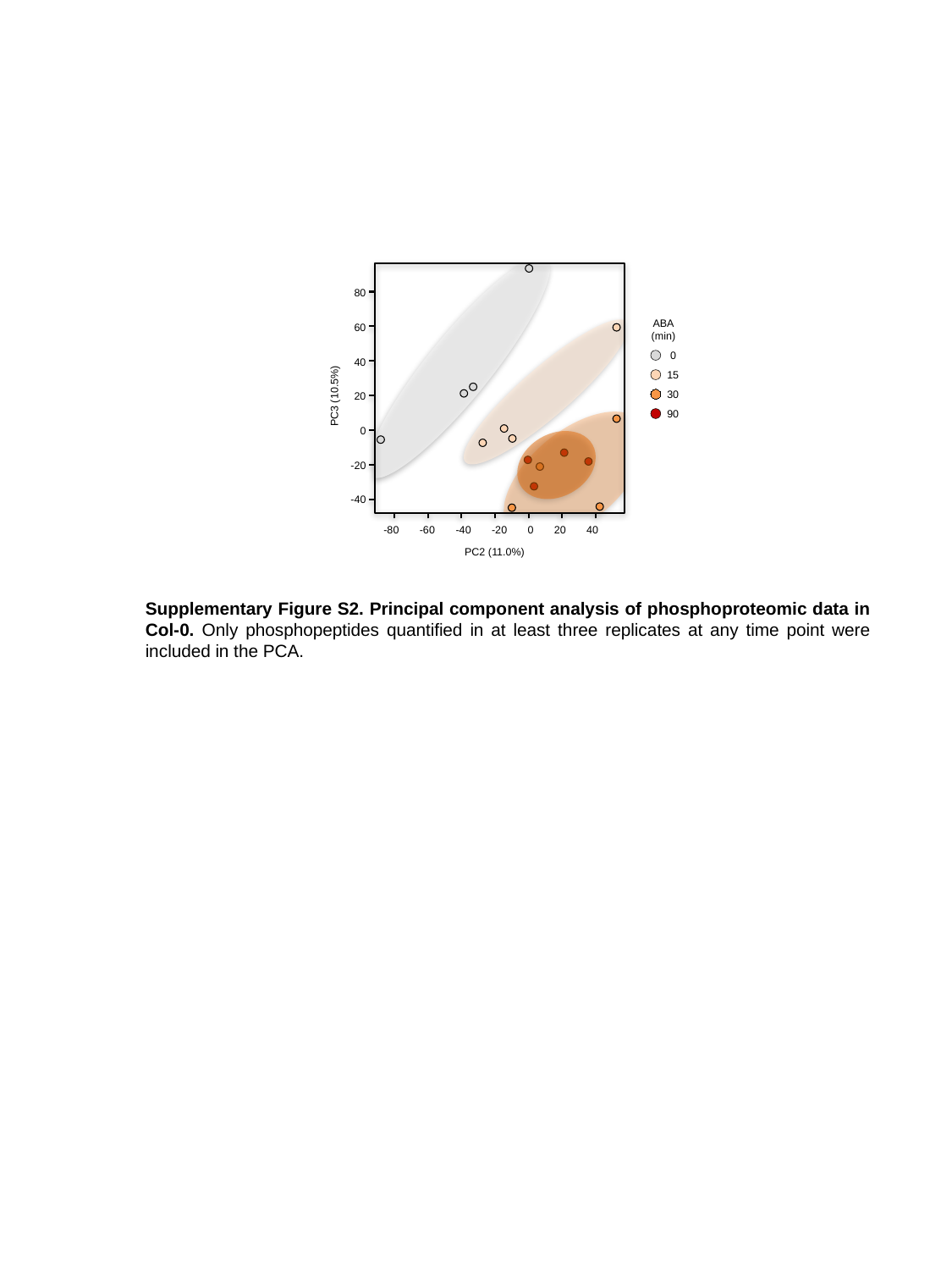

80
60
40
20
PC3 (10.5%)
0
-20
-40
-80
-60
-40
-20
0
20
40
PC2 (11.0%)
ABA
(min)
0
15
30
90
Supplementary Figure S2. Principal component analysis of phosphoproteomic data in Col-0. Only phosphopeptides quantified in at least three replicates at any time point were included in the PCA.

## Slide 3
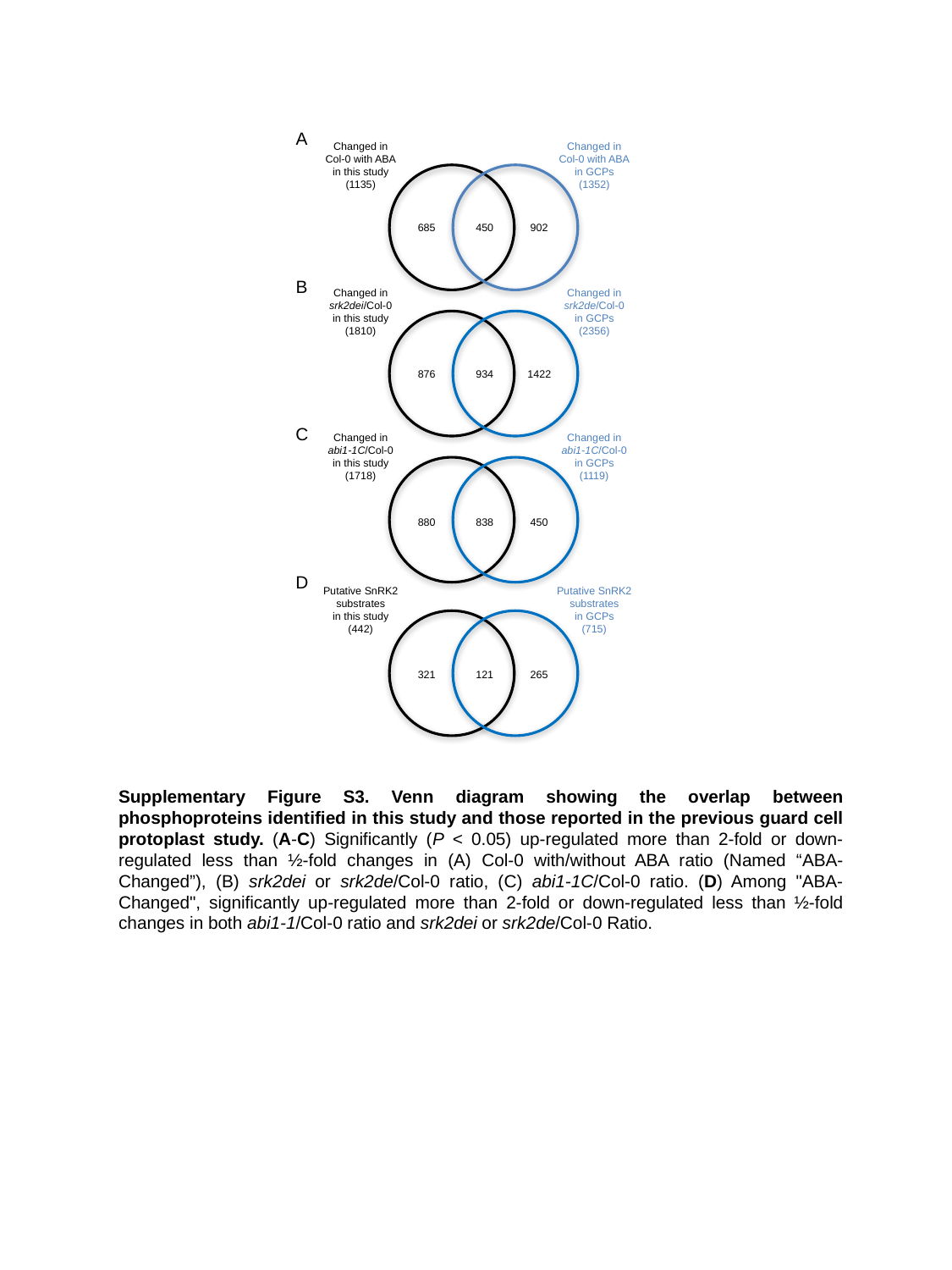

A
Changed in
Col-0 with ABA
in this study
(1135)
Changed in
Col-0 with ABA
in GCPs
(1352)
685
450
902
B
Changed in
srk2dei/Col-0
in this study
(1810)
Changed in
srk2de/Col-0
in GCPs
(2356)
876
934
1422
C
Changed in
abi1-1C/Col-0
in this study
(1718)
Changed in
abi1-1C/Col-0
in GCPs
(1119)
880
838
450
D
Putative SnRK2 substrates
in this study
(442)
Putative SnRK2 substrates
in GCPs
(715)
321
121
265
Supplementary Figure S3. Venn diagram showing the overlap between phosphoproteins identified in this study and those reported in the previous guard cell protoplast study. (A-C) Significantly (P < 0.05) up-regulated more than 2-fold or down-regulated less than ½-fold changes in (A) Col-0 with/without ABA ratio (Named “ABA-Changed”), (B) srk2dei or srk2de/Col-0 ratio, (C) abi1-1C/Col-0 ratio. (D) Among "ABA-Changed", significantly up-regulated more than 2-fold or down-regulated less than ½-fold changes in both abi1-1/Col-0 ratio and srk2dei or srk2de/Col-0 Ratio.

## Slide 4
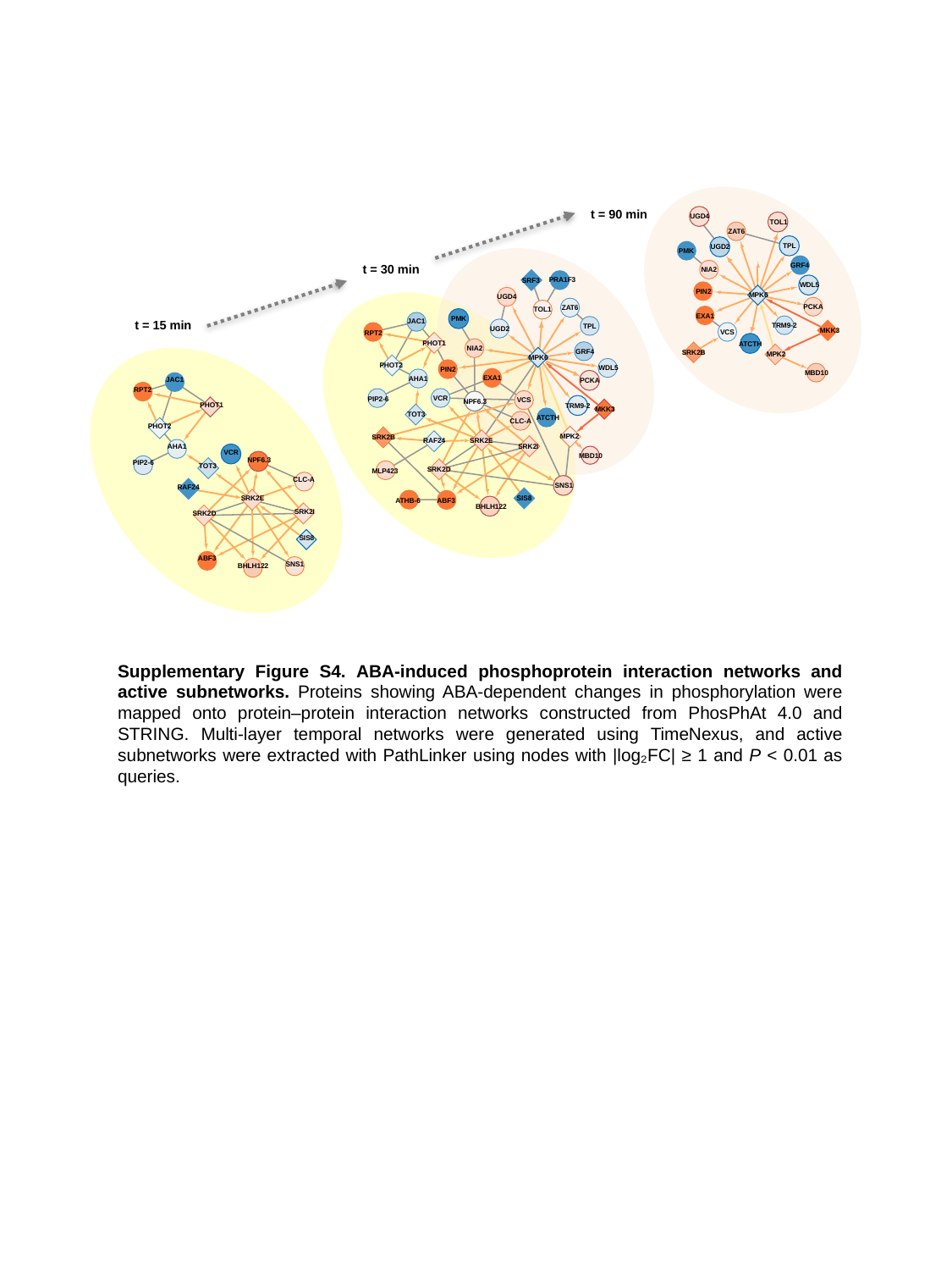

UGD4
TOL1
ZAT6
TPL
UGD2
PMK
GRF4
NIA2
WDL5
PIN2
MPK6
PCKA
EXA1
TRM9-2
MKK3
VCS
ATCTH
SRK2B
MPK2
MBD10
t = 90 min
PRA1F3
SRF3
UGD4
ZAT6
TOL1
PMK
JAC1
TPL
UGD2
RPT2
PHOT1
NIA2
GRF4
MPK6
PHOT2
WDL5
PIN2
EXA1
AHA1
PCKA
VCR
PIP2-6
VCS
NPF6.3
TRM9-2
MKK3
TOT3
ATCTH
CLC-A
MPK2
SRK2B
SRK2E
RAF24
SRK2I
MBD10
SRK2D
MLP423
SNS1
SIS8
ATHB-6
ABF3
BHLH122
t = 30 min
t = 15 min
JAC1
RPT2
PHOT1
PHOT2
AHA1
VCR
NPF6.3
PIP2-6
TOT3
CLC-A
RAF24
SRK2E
SRK2I
SRK2D
SIS8
ABF3
SNS1
BHLH122
Supplementary Figure S4. ABA‑induced phosphoprotein interaction networks and active subnetworks. Proteins showing ABA‑dependent changes in phosphorylation were mapped onto protein–protein interaction networks constructed from PhosPhAt 4.0 and STRING. Multi‑layer temporal networks were generated using TimeNexus, and active subnetworks were extracted with PathLinker using nodes with |log₂FC| ≥ 1 and P < 0.01 as queries.

## Slide 5
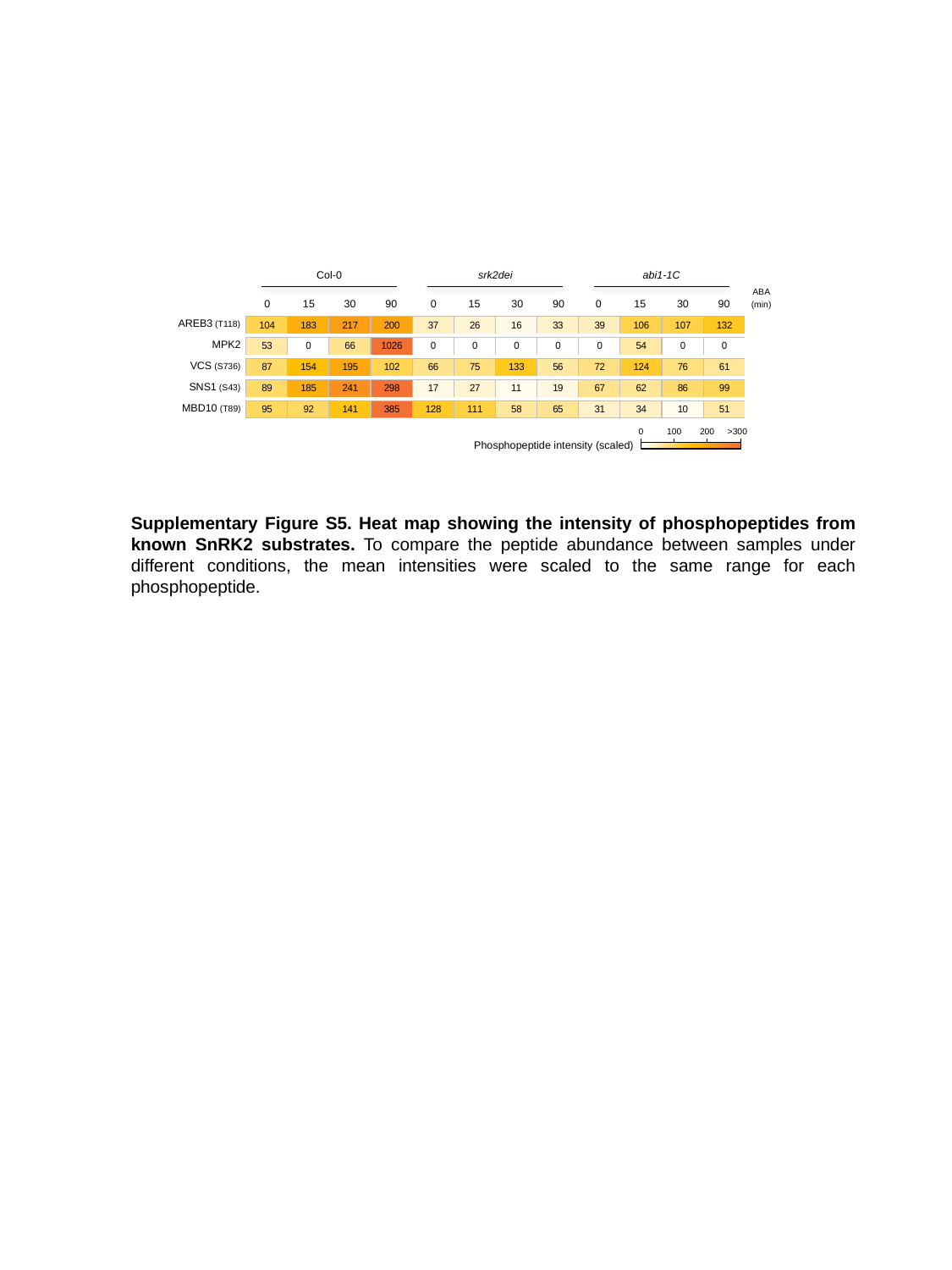

Col-0
srk2dei
abi1-1C
ABA
(min)
0
15
30
90
0
15
30
90
0
15
30
90
AREB3 (T118)
MPK2
VCS (S736)
SNS1 (S43)
MBD10 (T89)
0
100
200
>300
Phosphopeptide intensity (scaled)
Supplementary Figure S5. Heat map showing the intensity of phosphopeptides from known SnRK2 substrates. To compare the peptide abundance between samples under different conditions, the mean intensities were scaled to the same range for each phosphopeptide.

## Slide 6
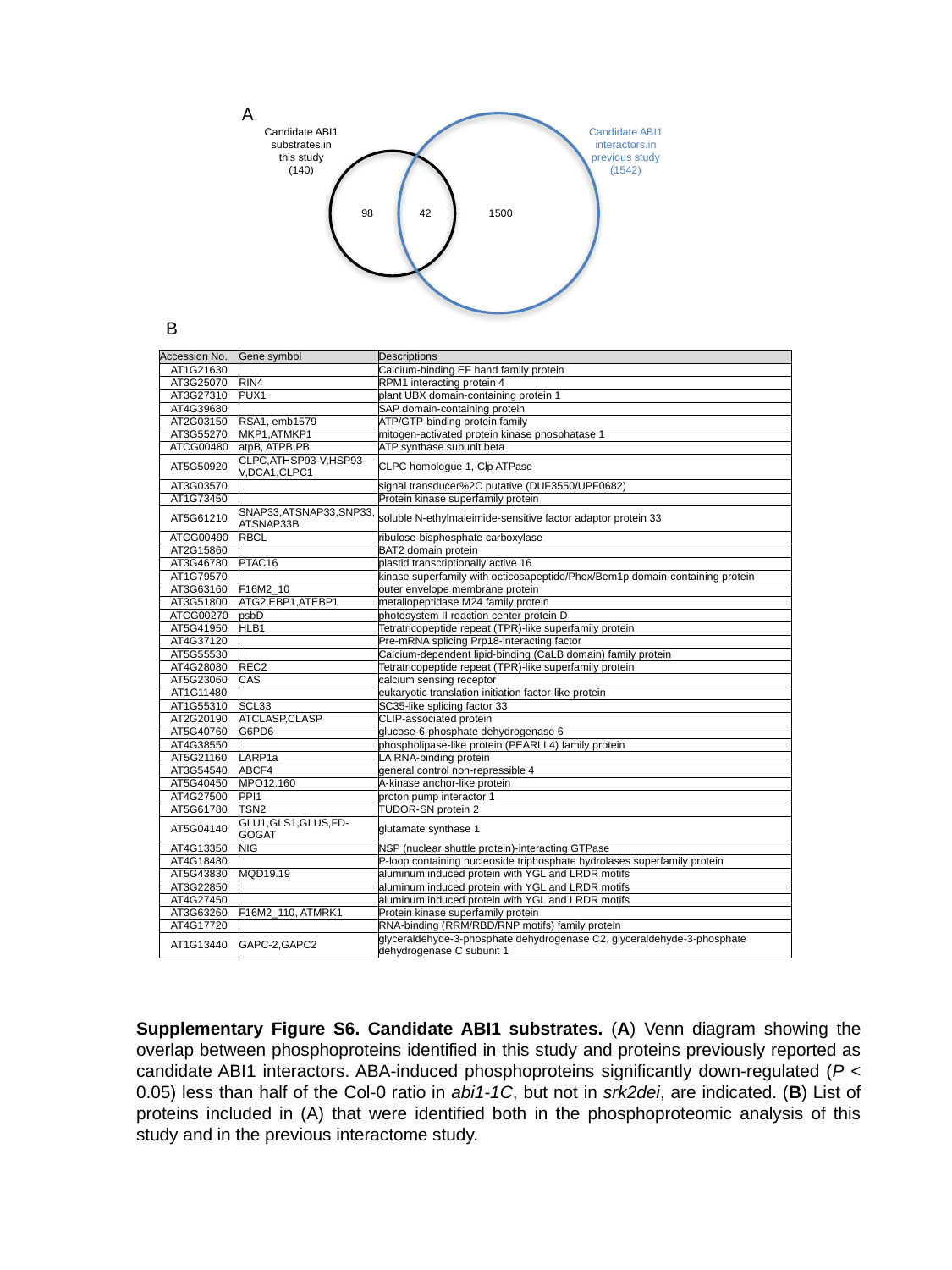

A
Candidate ABI1 substrates.in this study
(140)
Candidate ABI1 interactors.in previous study
(1542)
98
42
1500
B
| Accession No. | Gene symbol | Descriptions |
| --- | --- | --- |
| AT1G21630 | | Calcium-binding EF hand family protein |
| AT3G25070 | RIN4 | RPM1 interacting protein 4 |
| AT3G27310 | PUX1 | plant UBX domain-containing protein 1 |
| AT4G39680 | | SAP domain-containing protein |
| AT2G03150 | RSA1, emb1579 | ATP/GTP-binding protein family |
| AT3G55270 | MKP1,ATMKP1 | mitogen-activated protein kinase phosphatase 1 |
| ATCG00480 | atpB, ATPB,PB | ATP synthase subunit beta |
| AT5G50920 | CLPC,ATHSP93-V,HSP93-V,DCA1,CLPC1 | CLPC homologue 1, Clp ATPase |
| AT3G03570 | | signal transducer%2C putative (DUF3550/UPF0682) |
| AT1G73450 | | Protein kinase superfamily protein |
| AT5G61210 | SNAP33,ATSNAP33,SNP33,ATSNAP33B | soluble N-ethylmaleimide-sensitive factor adaptor protein 33 |
| ATCG00490 | RBCL | ribulose-bisphosphate carboxylase |
| AT2G15860 | | BAT2 domain protein |
| AT3G46780 | PTAC16 | plastid transcriptionally active 16 |
| AT1G79570 | | kinase superfamily with octicosapeptide/Phox/Bem1p domain-containing protein |
| AT3G63160 | F16M2\_10 | outer envelope membrane protein |
| AT3G51800 | ATG2,EBP1,ATEBP1 | metallopeptidase M24 family protein |
| ATCG00270 | psbD | photosystem II reaction center protein D |
| AT5G41950 | HLB1 | Tetratricopeptide repeat (TPR)-like superfamily protein |
| AT4G37120 | | Pre-mRNA splicing Prp18-interacting factor |
| AT5G55530 | | Calcium-dependent lipid-binding (CaLB domain) family protein |
| AT4G28080 | REC2 | Tetratricopeptide repeat (TPR)-like superfamily protein |
| AT5G23060 | CAS | calcium sensing receptor |
| AT1G11480 | | eukaryotic translation initiation factor-like protein |
| AT1G55310 | SCL33 | SC35-like splicing factor 33 |
| AT2G20190 | ATCLASP,CLASP | CLIP-associated protein |
| AT5G40760 | G6PD6 | glucose-6-phosphate dehydrogenase 6 |
| AT4G38550 | | phospholipase-like protein (PEARLI 4) family protein |
| AT5G21160 | LARP1a | LA RNA-binding protein |
| AT3G54540 | ABCF4 | general control non-repressible 4 |
| AT5G40450 | MPO12.160 | A-kinase anchor-like protein |
| AT4G27500 | PPI1 | proton pump interactor 1 |
| AT5G61780 | TSN2 | TUDOR-SN protein 2 |
| AT5G04140 | GLU1,GLS1,GLUS,FD-GOGAT | glutamate synthase 1 |
| AT4G13350 | NIG | NSP (nuclear shuttle protein)-interacting GTPase |
| AT4G18480 | | P-loop containing nucleoside triphosphate hydrolases superfamily protein |
| AT5G43830 | MQD19.19 | aluminum induced protein with YGL and LRDR motifs |
| AT3G22850 | | aluminum induced protein with YGL and LRDR motifs |
| AT4G27450 | | aluminum induced protein with YGL and LRDR motifs |
| AT3G63260 | F16M2\_110, ATMRK1 | Protein kinase superfamily protein |
| AT4G17720 | | RNA-binding (RRM/RBD/RNP motifs) family protein |
| AT1G13440 | GAPC-2,GAPC2 | glyceraldehyde-3-phosphate dehydrogenase C2, glyceraldehyde-3-phosphate dehydrogenase C subunit 1 |
Supplementary Figure S6. Candidate ABI1 substrates. (A) Venn diagram showing the overlap between phosphoproteins identified in this study and proteins previously reported as candidate ABI1 interactors. ABA‑induced phosphoproteins significantly down‑regulated (P < 0.05) less than half of the Col-0 ratio in abi1‑1C, but not in srk2dei, are indicated. (B) List of proteins included in (A) that were identified both in the phosphoproteomic analysis of this study and in the previous interactome study.
